# Factorial Structure of the Serbian Version of the Clinical Assessment Interview for Negative Symptoms – Evidence for Three Factors of Negative Symptoms

**DOI:** 10.3389/fpsyg.2020.570356

**Published:** 2020-10-26

**Authors:** Ivan Ristić, Stefan Jerotić, Mirjana Zebić, Bojana Savić, Vuk Vuković, Manuela Russo, Tatjana Voskresenski, Nikolina Jovanović, Nađa P. Marić

**Affiliations:** ^1^ Faculty of Medicine, University of Belgrade, Belgrade, Serbia; ^2^ Clinic for Psychiatry, Clinical Centre of Serbia, Belgrade, Serbia; ^3^ Special Hospital for Psychiatric Diseases ‘Dr Slavoljub Bakalović’, Psychiatry, Vršac, Serbia; ^4^ Unit for Social and Community Psychiatry – WHO Collaborating Centre for Mental Health Services Development, Queen Mary University of London, London, United Kingdom; ^5^ Institute of Mental Health, Psychiatry, Belgrade, Serbia

**Keywords:** assessment, psychosis, negative symptoms, schizophrenia, CAINS

## Abstract

**Introduction:** Negative symptoms are a common occurrence in patients with psychosis spectrum disorders. Previous analysis of the latent structure of the Clinical Assessment Interview for Negative Symptoms (CAINS) – which was developed to advance the assessment of negative symptomatology – showed two underlying sub-domains (Motivation and Pleasure; Expression). Recent findings indicate that a more complex structure might be more applicable.

**Aim:** To evaluate the psychometric properties of the Serbian version of the CAINS in a sample of outpatients (*N* = 67) with psychosis spectrum disorders.

**Materials and Methods:** Negative symptoms and general level of psychopathology were assessed with Serbian translations of the CAINS, the 53-item version of the Brief Symptom Inventory (BSI), and the 24-item version of the Brief Psychiatric Rating Scale (BPRS). Principal component analysis (PCA) was carried out on the CAINS items, and correlation analyses were done to assess its convergent and discriminant validity.

**Results:** Our results showed an excellent internal consistency (Cronbach’s alpha = 0.92). PCA revealed a three-component solution consisting of Expressiveness and Motivation for Social and Family Relationships (Factor 1), Motivation for Vocational Activities (Factor 2), and Motivation for Recreation (Factor 3). Convergent validity was supported by significant correlations with the Negative symptoms domain of the BPRS (Factor 1, 0.695, *p* < 0.01; Factor 2, 0.352, *p* < 0.05; Factor 3, 0.452, *p* < 0.01). When assessing discriminant validity, weak correlations were found with BPRS and BSI scores.

**Conclusion:** The Serbian version of CAINS is a valid, reliable and useful tool for the assessment of negative symptomatology. Our findings support a three-factor structure of CAINS, which indicates that the construct is more complex than envisaged by the original conceptualization of two distinct factors.

## Introduction

Previous factor analyses of symptoms related to schizophrenia revealed several clusters of symptoms, the most robust being positive and negative dimensions (Lenzenweger et al., 1989). Although these dimensions were evident in schizophrenia, they were also reported with lesser degrees of severity in the general population (Maric et al., 2004).

The importance of negative symptoms in psychosis can hardly be overstated. A recent multicenter study on the epidemiology of negative symptoms found at least one of the previously described symptoms in approximately 58% in an outpatient sample (Bobes et al., 2010). Furthermore, primary negative symptoms (primary as predating the onset of other psychotic symptoms) were found in around 13% of the patients (Bobes et al., 2010). Negative symptoms are not only common and intrinsically bound to the syndrome of schizophrenia, but also strikingly associated with functional outcomes. Severity of negative symptoms predicts the degree of impairment in relationships, work performance, and global psychosocial functioning (Milev et al., 2005). One study found that while adaptive life skills are mostly predicted by cognitive functioning, negative symptoms affected primarily interpersonal skills (Bowie et al., 2006). Both cognitive status and negative symptoms were significantly associated with functional outcome.

From their first clear conceptualization by Bleuler, negative symptoms have remained a diagnostically elusive category. These symptoms, sometimes called “the deficit syndrome,” have been conceived as the following five constructs: anhedonia, asociality, alogia, avolition, and blunted affect (developed from Bleuler’s “four A’s”; Moskowitz and Heim, 2011). In 2005, a panel of experts reached a consensus on negative symptoms that provided support for the abovementioned five domain conceptualization, although substantial correlations between the domains exist (Kirkpatrick et al., 2006). Even though these domains are intercorrelated, negative symptoms may have separate biological substrates. One study found that the five constructs cluster into two factors, one including blunted affect and alogia, and the other anhedonia, avolition, and asociality (Marder and Galderisi, 2017). However, another study reported that the conceptualization of the latent structure of negative symptoms as two distinct dimensions may not completely capture the complexity of the construct, and that the best fit was represented by the five consensus domains (anhedonia, asociality, avolition, blunted affect, and alogia; Strauss et al., 2018).

Historically, negative symptoms have been assessed using different instruments. The so-called first generation of instruments such as the Positive and Negative Syndrome Scale (PANSS; Kay et al., 1987), Scale for Assessment of Negative Symptoms (SANS; Andreasen, 1989), and Negative Symptom Assessment (NSA; Axelrod et al., 1993) have been criticized for their conceptual limitations (Couture et al., 2011), primarily by exploring associations between beliefs and global indices of negative symptoms rather than different negative symptom domains. In the consensus statement, Kirkpatrick et al. (2006) discuss some examples such as: items not reflecting current understanding of negative symptoms constructs, individual item ratings reflecting conceptually distinct processes (e.g., assessment of reduced emotional range in NSA including both anhedonia and lack of emotional experiences), and inconsistent use of behavioral vs. experiential referents (e.g., PANSS relies on observation of behavior during the interview and reports of behavior gained from family; Kirkpatrick et al., 2006).

In order to advance the quantification of negative symptoms and its use in both clinical and research practices, the Collaboration to Advance Negative Symptom Assessment in Schizophrenia developed the Clinical Assessment Interview for Negative Symptoms (CAINS; Horan et al., 2011). The scale was designed to assess the five negative domains based on the aforementioned consensus by Kirkpatrick et al. (2006), as well as to address the limitations of the previously used scales. The analysis of the latent structure of the CAINS showed two underlying factors which reflect experiential (Motivation and Pleasure) and expressive aspects (Expression; Kring et al., 2013). Experiential determinants include: diminished motivation and engagement in pleasurable social, vocational, and recreational activities. Expressive determinants are comprised of reduced verbal and non-verbal communicative output. The CAINS instrument has since been translated and validated in several countries (Engel et al., 2014; Chan et al., 2015; Valiente-Gómez et al., 2015; Jung et al., 2016).

To the best of our knowledge, this is the first validation of CAINS in Serbia. Furthermore, no instruments that measure negative symptomatology have been validated on Serbian patients. The aim of this study was to evaluate the factor structure, validity, and reliability of the Serbian translation of CAINS on a sample of remitted outpatients with a confirmed diagnosis of psychosis spectrum disorders.

## Materials And Methods

### Participants and Procedure

The study included 67 patients with psychosis spectrum disorders, consecutively recruited from two outpatient clinics in Serbia (University Clinic for Psychiatry, Clinical Centre of Serbia in Belgrade and Special Hospital for Psychiatric Diseases “Dr Slavoljub Bakalovic” in Vrsac). Inclusion criteria were: primary diagnosis of psychosis or related disorder (ICD-10 F20–F29) in remission, over 18 years of age, attending the outpatient clinic or day hospital, history of at least one hospital admission in their lifetime, no plans of being discharged from mental health care services for the next 12 months, and capacity to provide informed consent. Exclusion criteria were: confirmed diagnosis of an organic brain disorder and presence of severe cognitive deficits, which rendered the individual unable to complete the interview (based on clinical judgment). All of the diagnoses were made by clinical psychiatrists treating the patients. All participants underwent a structured socio-demographic interview collecting information about age, sex, marital status, employment status, and highest level of educational achievement.

The study was conducted in accordance with the Declaration of Helsinki and its design was approved by the Medical Ethics Committee of the Faculty of Medicine University of Belgrade, as well as by the professional boards of both study sites. All participants provided written informed consent before the study. This study was done as part of the Horizon2020 “Implementation of an effective and cost-effective intervention for patients with psychotic disorders in low‐ and middle-income countries in South Eastern Europe” (IMPULSE) project funded by the European Commission. More information about the project’s clinical study is available in the published protocol (Jovanovic et al., 2019).

### Instruments of Measurement

The CAINS is a 13-item interview that is used to assess the presence and severity of negative symptoms (Kring et al., 2013). All items are scored on a five-point scale ranging from 0 (no impairment) to 4 (severe impairment). CAINS assesses motivation and pleasure for social, work, and recreational activities, and also assesses emotional expression.

The latest version of CAINS and the CAINS manual were translated into Serbian by a research assistant and then back-translated to English by a translator who had not previously seen the original version. After discussion with the translators and the members of the research team, a version of the Serbian CAINS was finalized (Supplementary Materials).

Training of the study researchers to use CAINS started with review of the manual and scoring guidelines. Subsequently, four researchers independently rated three videotaped interviews provided by the developers of CAINS. Once ratings were completed, researchers engaged in a comprehensive discussion, including comparing their ratings with the gold standard ratings given by the developers. A final excellent inter-rater reliability was achieved (Intraclass Correlation Coefficient = 0.98).

The 24-item version of the Brief Psychiatric Rating Scale (BPRS) was used to determine the symptomatology of the participants. Principal component analysis (PCA) of the 24-item BPRS in previous research indicated a four-factor solution, where each factor comprised of several items from the BPRS: Manic-excitement (Motor hyperactivity, Elevated mood, Excitement, Distractibility, Hostility, and Grandiosity), Negative symptoms (Blunted affect, Motor retardation, Emotional withdrawal, and Self-neglect), Positive symptoms (Bizarre behavior, Unusual thought content, Disorientation, Hallucinations, and Suspiciousness), and Depression-anxiety (Depression, Anxiety, Suicidality, and Guilt; Ventura et al., 2000).

The 53-item version of the Brief Symptom Inventory (BSI; Derogatis and Melisaratos, 1983) was used to evaluate the overall levels of psychopathology. The BSI consists of nine primary symptom dimensions as follows: Somatization, Obsession-Compulsion, Interpersonal Sensitivity, Depression, Anxiety, Hostility, Phobic Anxiety, Paranoid Ideation, and Psychoticism.

### Data Analysis

In order to assess construct validity, a PCA of the Serbian CAINS was carried out. The Kaiser-Meyer-Olkin test for sampling adequacy and the Bartlett’s Test of Sphericity were used to measure sampling adequacy for each variable in the model. The promax rotation method, assuming intercorrelation between factors, was used. To determine the number of factors to retain, eigenvalues higher than 1.0 and inspection of the scree plot were used. This number was further evaluated using the parallel analysis matrix. A symptom-to-factor loading cut off point of ±0.4 was used to interpret the individual item loadings (Boyd, 2013). For items in which cross-loading was present, the factor on which the item loaded higher was chosen. Internal consistency was determined by calculating Cronbach’s alpha for the whole scale as well as all separate factors. Correlational analysis was done to investigate the convergent and discriminant validity of CAINS subscales. Convergent validity was evaluated based on the correlations with the BPRS Negative symptoms factor. Discriminant validity was evaluated through correlations with the nine BSI symptom dimensions, BSI total score, BPRS Depression-anxiety and Positive symptoms factors, and BPRS total score.

## Results

### Sociodemographic and Clinical Characteristics of the Sample

The sample size was considered adequate for PCA based on the recommended minimum participant-to-item ratio of 5:1 (Gorsuch, 1990) with the total number of participants (*n* = 67) exceeding the recommended value of 65 (five times the number of 13 questionnaire items). The sample consisted of 60.9% male participants, with a mean age of 43.6 ± 10.9 (age range of 22–68). Detailed sociodemographic and clinical characteristics of the sample are given in Table 1.

**Table 1:** Sociodemographic characteristics and clinical measures.

	Study participants *N* = 67 (100%)
Age, years; mean (SD); Range (min-max)	43.6 (10.9); 22–68
Duration of illness, years; mean (SD); Range (min-max)	13.2 (9.6); 1–35
Gender, N (%)
Male	41 (60.9)
Marital status, N (%)
Single	51 (76.1)
In a relationship/married	8 (11.9)
Divorced	8 (11.9)
Employment, N (%)
Unemployed	33 (49.3)
Employed	7 (10.4)
Retired	27 (37.3)
Education, N (%)
Elementary school	4 (6.0)
High school	53 (79.1)
College/University	10 (14.9)
Clinical diagnosis ICD-10, N (%)
F20 – Schizophrenia	22 (32.8)
F22 – Persistent delusional disorders	4 (6.0)
F23 – Acute and transient psychotic disorders	5 (7.6)
F25 – Schizoaffective disorders	11 (19.3)
F29 – Unspecified nonorganic psychosis	25 (37.3)
Clinical measures, mean (SD)
BPRS total score	1.94 ± 0.73
BPRS Negative symptoms	2.20 ± 0.94
BPRS Depression-anxiety	2.46 ± 0.99
BPRS Manic-excitement	1.58 ± 0.46
BPRS Positive symptoms	1.42 ± 0.61
BSI Total score	1.17 ± 0.82
BSI Depression	0.97 ± 0.60
BSI Somatization	0.77 ± 0.71
BSI Obsession-compulsion	1.32 ± 0.96
BSI Interpersonal sensitivity	1.33 ± 0.94
BSI Anxiety	1.02 ± 0.78
BSI Hostility	0.47 ± 0.54
BSI Phobic anxiety	0.89 ± 0.81
BSI Paranoid ideation	1.13 ± 0.91
BSI Psychoticism	0.86 ± 0.71

BPRS, Brief Psychiatric Rating Scale; BSI, Brief Symptom Inventory; ICD-10 = 10th revision of the International Statistical Classification of Diseases and Related Health Problems; SD, standard deviation.

### The CAINS Questionnaire Structure

**Figure 1:**
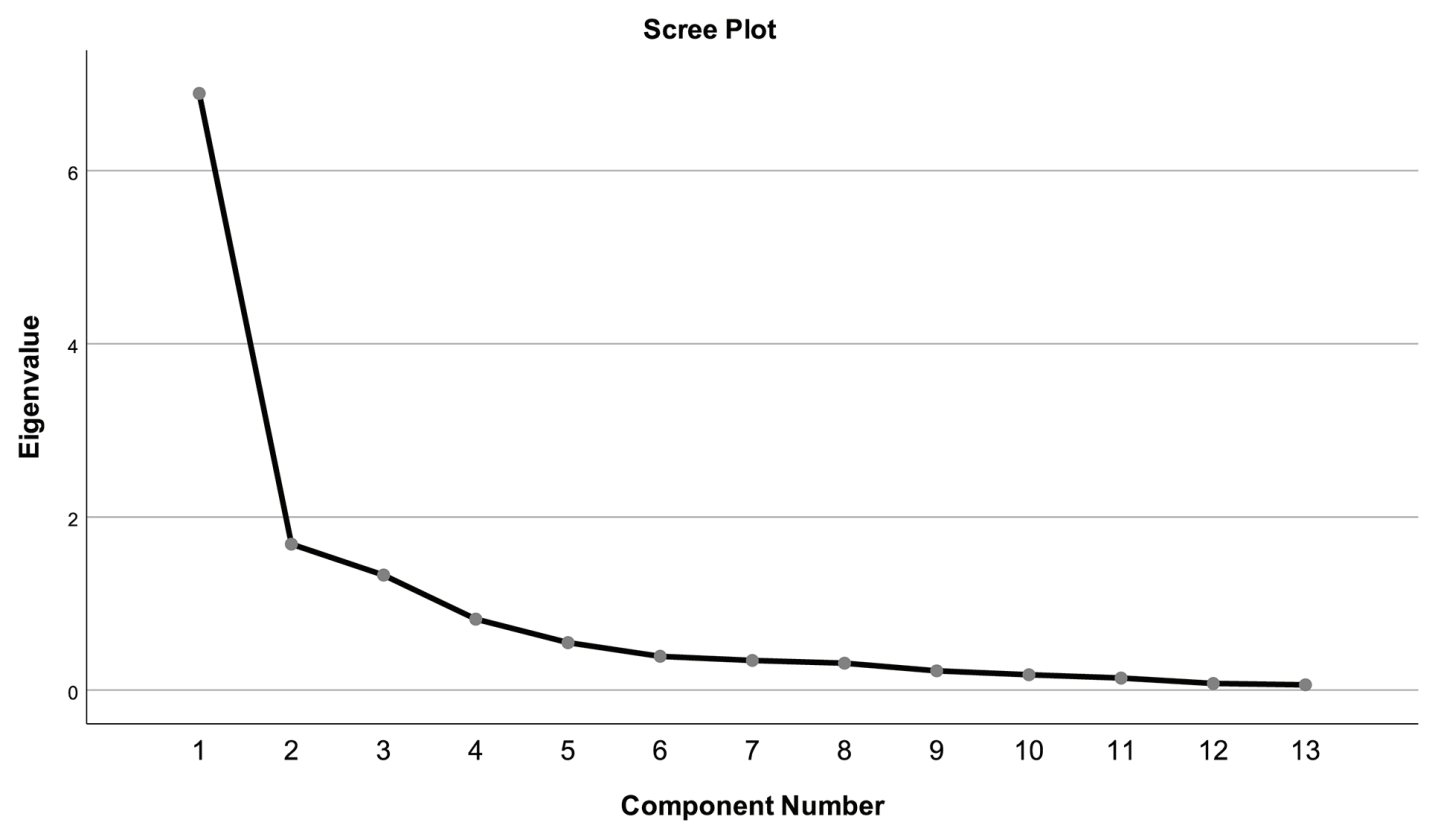
Scree plot representing the eigenvalues received through principal component analysis.

The 13 items of the CAINS were subjected to PCA (Table 2). Sampling adequacy was ascertained by a Kaiser-Meyer-Olkin test result value equal to 0.829, thus exceeding the recommended value of 0.6, and by the Bartlett’s Test of Sphericity (*p* < 0.001). The PCA revealed the presence of three factors (Table 2). The total amount of variance explained was 76.21%, with each factor accounting for the following percentage (and eigenvalues) 53.02% (6.893), 12.97%, (1.687), and 10.22% (1.329), respectively. Inspection of the scree plot confirmed the presence of three factors (Figure 1). The results of parallel analysis showed three factors with eigenvalues exceeding the corresponding criterion values for a randomly generated data matrix of the same size (13 variables × 67 patients). Based on factor loading on each of the obtained factors, the first factor could be labeled as “Expressiveness and Motivation for Social and Family Relationships,” the second factor as “Motivation for Recreation,” and the third factor as “Motivation for Vocational Activities.” The factor correlation matrix is shown in Table 3. The factors had low to moderate intercorrelations.

**Table 2:** Three-factor solution for the Clinical Assessment Interview for Negative Symptoms (CAINS) items.

CAINS item	Factor 1	Factor 2	Factor 3
Item 3: Social, past-week pleasure	0.839	0.599	0.211
Item 4: Social, expected pleasure	0.830	0.629	0.197
Item 10: Expression, facial	0.814	0.284	0.586
Item 11: Expression, vocal prosody	0.796	0.250	0.549
Item 13: Expression, speech	0.757	0.140	0.648
Item 12: Expression, gestures	0.755	0.161	0.681
Item 1: Social, family relationships	0.745	0.502	0.187
Item 2: Social, friendships	0.721	0.399	0.282
Item 9: Recreation, expected pleasure	0.453	0.922	0.315
Item 8: Recreation, past-week pleasure	0.397	0.913	0.295
Item 7: Recreation, motivation	0.507	0.882	0.391
Item 5: Vocational, motivation	0.396	0.319	0.902
Item 6: Vocational, expected pleasure	0.453	0.448	0.859

Factor 1 = Expressiveness and Motivation for Social and Family Relationships; Factor 2 = Motivation for Recreational; Factor 3 = Motivation for Vocational Activities.

**Table 3:** Factor transformation matrix.

	Factor 1	Factor 2	Factor 3
Factor 1	..	..	..
Factor 2	0.431	..	..
Factor 3	0.448	0.185	..

Factor 1 = Expressiveness and Motivation for Social and Family Relationships; Factor 2 = Motivation for Recreational; Factor 3 = Motivation for Vocational Activities.

Cronbach’s alpha coefficient for the entire CAINS was 0.92. All of the separate factors exceeded the recommended 0.70 cutoff point for Cronbach’s alpha (Bland and Altman, 1997), showing excellent internal consistency. Item and separate factor statistics are given in Table 4.

**Table 4:** Item scores, factor scores, and internal consistency of the CAINS.

	Mean	SD	Cronbach’s alpha
Factor 1	13.07	6.17	0.910
Item 1: Social, family relationships	1.48	0.88	
Item 2: Social, friendships	2.09	1.11	
Item 3: Social, past-week pleasure	2.19	0.94	
Item 4: Social, expected pleasure	2.27	1.02	
Item 10: Expression, facial	1.37	0.10	
Item 11: Expression, vocal prosody	1.31	0.87	
Item 12: Expression, gestures	1.22	1.06	
Item 13: Expression, speech	1.13	0.97	
Factor 2	5.40	3.20	0.939
Item 7: Recreation, motivation	1.85	1.13	
Item 8: Recreation, past-week pleasure	1.70	1.09	
Item 9: Recreation, expected pleasure	1.85	1.17	
Factor 3	5.56	2.20	0.913
Item 5: Vocational, motivation	2.48	1.25	
Item 6: Vocational, expected pleasure	3.09	1.04	
CAINS total score	24.04	9.80	0.920

Factor 1 = Expressiveness and Motivation for Social and Family Relationships; Factor 2 = Motivation for Recreational; Factor 3 = Motivation for Vocational Activities; SD, standard deviation.

### Convergent and Discriminant Validity

All three CAINS factors correlated with the Negative symptoms BPRS factor, thus indicating good convergent validity. When assessing discriminant validity, we found that the Factor 3 did not correlate with BPRS or with BSI, while Factors 1 and 2 showed small but significant correlations ranging from 0.261 (BSI total) to 0.493 (BPRS Total; Table 5).

**Table 5:** Convergent and discriminant validity of the CAINS.

	CAINS total	Factor 1	Factor 2	Factor 3
BPRS Negative symptoms	0.798^**^	0.695^**^	0.352^*^	0.452^**^
BPRS Depression-anxiety	0.280^*^	0.217	0.301^*^	0.199
BPRS Positive symptoms	0.307^*^	0.304^*^	0.273^*^	0.333
BPRS Total	0.493^*^	0.486^*^	0.422^**^	0.356
BSI Depression	0.329^**^	0.283^*^	0.299^*^	0.238
BSI Somatization	0.202	0.195	0.183	0.086
BSI Obsession-compulsion	0.212^*^	0.299^*^	0.189	0.181
BSI Interpersonal sensitivity	0.288^*^	0.260^*^	0.250^*^	0.192
BSI Anxiety	0.099	0.080	0.118	0.043
BSI Hostility	0.069	0.035	0.236	0.059
BSI Phobic anxiety	0.189	0.182	0.232	0.006
BSI Paranoid ideation	0.101	0.108	0.130	0.040
BSI Psychoticism	0.265^*^	0.289^*^	0.156	0.140
BSI Total	0.261^*^	0.242^*^	0.246^*^	0.128

* *p* < 0.05

** *p* < 0.01.

Factor 1 = Expressiveness and Motivation for Social and Family Relationships; Factor 2 = Motivation for Recreational; Factor 3 = Motivation for Vocational Activities.

## Discussion

The results of our study validate the Serbian version of the CAINS in a sample of outpatients with ICD-10 diagnoses F20–F29, including a spectrum of psychotic disorders broader than schizophrenia. The demographic characteristics of our sample were similar to those in studies that originally validated the CAINS (Forbes et al., 2010; Horan et al., 2011; Kring et al., 2013). Our sample comprised people with established illness (average illness duration of 13 years), and the scores on BPRS were somewhat higher than in the original validation of CAINS, indicating a more severe psychopathology (Kring et al., 2013).

Our results suggest that the Serbian version of the CAINS is better described by three factors that, based on their item loading, can be labeled as follows: Expressiveness and Motivation for Social and Family Relationships (Factor 1), Motivation for Recreation (Factor 2), and Motivation for Vocational Activities (Factor 3).

The three-factor structure was found to be different from the original CAINS validation (Kring et al., 2013), as well as from validations done in other languages (Engel et al., 2014; Chan et al., 2015; Valiente-Gómez et al., 2015; Jung et al., 2016), which mostly found a two-factor structure (“Motivation and Pleasure” and “Expression”). A recent cross-cultural confirmatory factor analysis found that a hierarchical model, in which motivational and expressive second-order factors are included with the five first-order factors (alogia, anhedonia, avolition, asociality, and blunted affect), has equally good fit as a model which includes only the first-order factors (Ahmed et al., 2019). Considering that Garcia-Portilla et al. (2014) found that a three-factor structure might better separate the multidimensional construct of negative symptoms than a two-factor structure, it is possible that the previous two-factor structure consensus might be premature.

In our structure, the first factor, Expressiveness and Motivation for Pleasure for Social and Family Relationships, combines expressiveness and motivational aspects. A study by Del-Monte et al. (2013) evaluating nonverbal expressive behavior found that there was a significant correlation of poor social functioning with decreased expressive behavior for patients with schizophrenia. Moreover, although this factor is different from what has been found in other studies, the aspects of expressiveness and motivation are interconnected, and the degree of expression may be crucial for maintaining social relationships. These aspects are tightly related as both represent core features of social cognition, which is not only known to be impaired in schizophrenia and related disorders (Green et al., 2015), but is also known to be associated with negative symptoms. Specifically, a recent conceptualization about negative symptoms and social cognition considers the motivational aspect crucial in the interface between social abilities and negative symptoms (Green, 2020), thus contributing to the impact of negative symptoms on the level of functioning.

The other two factors were separated into Motivation for Vocational activities and Motivation for Recreation, which might underpin two different sets of motivational behaviors in relation to ludic and recreational activities as opposed to professional and work-oriented activities.

Reliability of the Serbian CAINS was supported by excellent internal consistency (Cronbach alpha > 0.9) of the whole scale and of item loading of the different factors. The original validation of the CAINS reported slightly lower Cronbach’s alpha for the whole CAINS scale (0.76).

Convergent validity of CAINS toward the BPRS Negative symptoms domain was found in our sample for the total CAINS score, as well as the separate factors. However, when evaluating discriminant validity, weak correlations were found between the CAINS total score and BPRS and BSI total scores, as well as BPRS Depression-anxiety and Positive Symptoms factors and the BSI Depression symptom domain. It has been found that some depressive features and negative symptoms in schizophrenia still continue to overlap. It is well known that (Krynicki et al., 2018) anhedonia, anergia, and avolition may be common to both depressive and negative symptoms in patients with schizophrenia. This could explain the weak correlation when evaluating discriminant validity in our sample. A similar finding was seen in the original CAINS validation (Kring et al., 2013), where the BPRS Positive Symptoms factor had a correlation of 0.31 with the CAINS Motivation/Pleasure subscale. The relationship between positive, negative, and depressive symptoms in psychotic disorders is complex, and certain overlap can be expected. Using latent variable structural equation modeling, two recent studies on large samples (Carrà et al., 2019, 2020) found that positive and negative symptoms had a significant correlation both on 6‐ and 12-month follow-ups, even when accounting for depression as a mediating factor.

This study has several strengths. This was the first study validating a negative symptomatology scale done in Serbia. The sample size was adequate, and the design was coherent with clear inclusion and exclusion criteria. We used parallel analysis to improve the methodology through which the number of factors was received.

There are, however, some limitations. Even though our sample size was sufficient for PCA, a more conservative approach would consider a 10:1 ratio between the number of participants and number of items. Our three-factor structure was supported by moderate intercorrelations between the three factors and several items presented loadings over 0.40 on Factor 1, which is most likely a consequence of our sample size (Hair et al., 2009). Clinical diagnosis was not confirmed through a structured interview and the diagnoses encompassed a broader spectrum of psychotic disorders, compared to only schizophrenia in other CAINS validation papers.

Serbian CAINS was assessed at one time-point, which is why test-retest reliability of the instrument could not be established. The BPRS negative factor does not include alogia, anhedonia, or asociality/social withdrawal, and might therefore not reflect the most suitable measure of negative symptomatology to determine convergent validity. Using other questionnaires (such as SANS) that solely evaluate negative symptomatology could potentially improve our understanding of convergent validity and is a consideration for future research. Lastly, the study did not assess extrapyramidal syndrome which could potentially mask negative symptomatology.

In conclusion, the validity and reliability of the Serbian version of the CAINS were comparable to those of the original instrument version (Kring et al., 2013). Certain differences between the two versions may reflect some cultural specificities of our clinical population in interpreting and expressing negative symptoms. As a whole, the Serbian version of the CAINS is a valid, reliable, and useful tool for the assessment of negative symptomatology which can be used to advance the assessment and treatment of patients with negative symptoms in Serbia.

## Data Availability Statement

The raw data supporting the conclusions of this article will be made available by the authors, without undue reservation.

## Ethics Statement

The studies involving human participants were reviewed and approved by the Medical Ethics Committee of the Faculty of Medicine University of Belgrade, as well as by the professional boards of both study sites. The patients/participants provided their written informed consent to participate in this study.

## Author Contributions

IR was involved in all stages of manuscript creation. SJ, MZ, BS, and VV were involved in data gathering, initial draft and final version revisions. MR and NJ were involved in statistical analysis, English language checks, and all stages of manuscript writing. TV was involved in initial draft and final version revisions. NM was involved in statistical analysis, statistical analyses and data interpretation, and all stages of manuscript writing. All authors contributed to the article and approved the submitted version.

### Conflict of Interest

The authors declare that the research was conducted in the absence of any commercial or financial relationships that could be construed as a potential conflict of interest.
