# Supplementary material for: Factorial Structure of the Serbian Version of the Clinical Assessment Interview for Negative Symptoms – Evidence for Three Factors of Negative Symptoms
Source: Front Psychol. 2020 Oct 26;11:570356. doi: 10.3389/fpsyg.2020.570356 (PMC7649283; doi:10.3389/fpsyg.2020.570356)
Supplement: Supplementary file 1 [file Data_Sheet_1.pdf]

Clinical Assessment Interview for Negative Symptoms (CAINS v1.0)

0 = Nema deficita
1 = Blagi deficit
2 = Umeren deficit
3 = Umereno ozbiljan deficit
4 = Ozbiljan deficit

SOCIJALNE RELACIJE (MOTIVACIJA I ZADOVOLJSTVO)

Q1: Motivacija za uspostavljanje bliskih porodičnih/bračnih/partnerskih Odnosa ☐

Q2: Motivacija za uspostavljanje bliskih prijateljskih i emotivnih veza ☐

Q3: Učestalost očekivanih ugodnih socijalnih aktivnosti – prethodna nedelja ☐

Q4: Učestalost očekivanih ugodnih socijalnih aktivnosti – naredna nedelja ☐

POSAO I OBRAZOVANJE (MOTIVACIJA I ZADOVOLJSTVO)

Q5: Motivacija za rad i školske aktivnosti ☐

Q6: Učestalost očekivanih ugodnih socijalnih aktivnosti – naredna nedelja ☐

REKREACIJA (MOTIVACIJA I ZADOVOLJSTVO)

Q7: Motivacija za razonodu (rekreativne aktivnosti) ☐

Q8: Učestalost prijatnih rekreativnih aktivnosti – prethodna nedelja ☐

Q9: Učestalost očekivanih ugodnih socijalnih aktivnosti – naredna nedelja ☐

EKSPRESIJA

Q10: Facijalna ekspresija ☐

Q11: Vokalna ekspresija ☐

Q12: Ekspresija pokreta ☐

Q13: Obim govora ☐

☐
