# Supplementary material for: Factorial Structure of the Serbian Version of the Clinical Assessment Interview for Negative Symptoms – Evidence for Three Factors of Negative Symptoms
Source: Front Psychol. 2020 Oct 26;11:570356. doi: 10.3389/fpsyg.2020.570356 (PMC7649283; doi:10.3389/fpsyg.2020.570356)
Supplement: Supplementary file 2 [file Data_Sheet_2.pdf]

Clinical Assessment Interview for Negative Symptoms (CAINS v1.0)

0 = No impairment
1 = Mild deficit
2= Moderate deficit
3 = Moderately severe deficit
4 = Severe deficit

SOCIAL: MOTIVATION & PLEASURE

Q1: Motivation for close family/spouse/partner relationships ☐

Q2: Motivation for close friendships & romantic relationships ☐

Q3: Frequency of pleasurable social activities – *past* week ☐

Q4: Frequency of expected pleasure from social activities – *next* week ☐

WORK & SCHOOL (MOTIVATION & PLEASURE)

Q5: Motivation for work & school activities ☐

Q6: Frequency of expected pleasure from work & school Activities - *next* week ☐

RECREATION (MOTIVATION & PLEASURE)

Q7: Motivation for recreational activities ☐

Q8: Frequency of pleasurable recreational activities – *past* week ☐

Q9: Frequency of expected pleasurable recreational activities – *next* week ☐

EXPRESSION

Q10: Facial expression ☐

Q11: Vocal expression ☐

Q12: Expressive gestures ☐

Q13: Quantity of speech ☐
